# Epidemic typhus imported from Algeria.

**DOI:** 10.3201/eid0505.990515

**Published:** 1999

**Authors:** M Niang, P Brouqui, D Raoult

**Affiliations:** Hopital Felix Houphoüet Boigny, Marseilles, France.

## Abstract

We report epidemic typhus in a French patient returning from Algeria. The diagnosis was confirmed by serologic testing and the isolation of Rickettsia prowazekii in blood. Initially the patient was thought to have typhoid fever. Because body lice are prevalent in industrialized regions, the introduction of typhus to pediculosis-endemic areas poses a serious public health risk.


### Epidemic Typhus Imported from Algeria

M. Niang,* P. Brouqui,*† and D. Raoult†
*Hopital Felix Houphoüet Boigny, Marseilles, France; and †Universitè de la Mèditerranèe, Facultè De Mèdecine, Marseilles, France

We report epidemic typhus in a French patient returning from Algeria. The diagnosis was confirmed by serologic testing and the isolation of *Rickettsia prowazekii* in blood. Initially the patient was thought to have typhoid fever. Because body lice are prevalent in industrialized regions, the introduction of typhus to pediculosis-endemic areas poses a serious public health risk.

Epidemic typhus, caused by *Rickettsia prowazekii*, is transmitted in the feces of the infected body louse. Body lice live in clothing and are easily controlled by good personal hygiene. Louse-infested populations are primarily those who live in extreme poverty. Typhus has been associated with war, famine, refugee camps, cold weather, and conditions that lead to domestic crowding and reduced personal hygiene, like those found in Algeria because of the ongoing civil war (1,2).

In October 1998, a 65-year-old man came to the Felix Houphoüet Boigny Hospital of Tropical Medicine in Marseilles, France, for evaluation of fever, nausea, vomiting, myalgias, and diarrhea. He was a native Algerian, who lived in France but had visited Msila, a small town in east central Algeria, for 3 months. The patient recalled pruritis and scratching during his stay in Algeria. However, no lice were found on his clothing. He had a temperature of 40°C, relative bradycardia with a heart rate of 70 beats per minute, blood pressure of 110/60 mm Hg, mild confusion, a discrete rash (a few rose spots on the trunk) (Figure 1), and splenomegaly. Laboratory findings showed a normal white blood cell count and increased serum lactate dehydrogenase (LDH 1032 IU/L) and aspartate aminotransferase (AST 85 IU/L) concentrations. The chest radiograph was normal. A presumptive diagnosis of typhoid fever was made, and treatment with intravenous ceftriaxone, 3 g/day (3), was started immediately after samples of blood, urine, and stool were obtained for bacterial cultures. All cultures remained negative on hospital day 3, but the patient's clinical condition continued to worsen. He had now become semicomatose, remained febrile, and had severe dyspnea and purpuric rash (Figure 2).

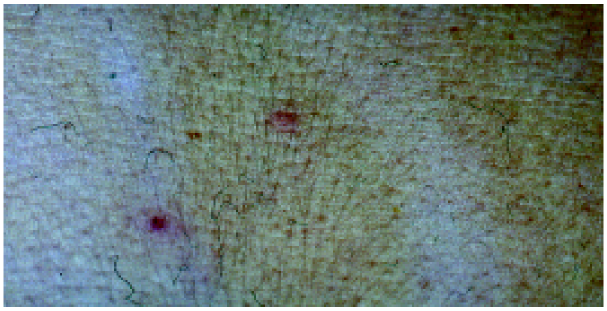

Figure 1. Rose spots-like skin lesion on the trunk (day 4).

Laboratory follow-up showed that the patient had severe thrombocytopenia (4,900

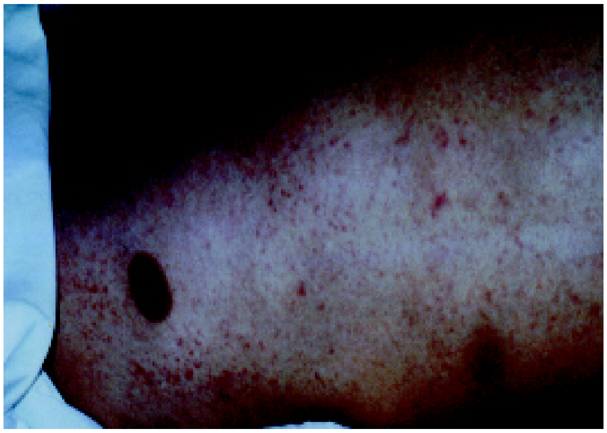

Figure 2. Diffuse petechial rash of epidemic typhus (day 7).

Address for correspondence: D. Raoult, Unitè des Rickettsies, CNRS UPRES A 6020, Universitè de la Mèditerranèe, Facultè de Mèdecine, 27 Boulevard Jean Moulin, 13385 Marseilles Cedex 05, France; fax: 334-91-83-0390; e-mail: Didier.Raoult@medecine.univ-mrs.fr.

thrombocytes/mm$^3$), and elevated serum creatinine phosphokinase (CK 1,4813 IU/L), AST (422 IU/L), LDH (2,373 IU/L), and creatinine (143 micromoles/L) concentrations. Coagulation studies suggested disseminated intravascular coagulation and acute renal failure (serum creatinine 143 micromoles/L). The patient was severely hypoxic, and a repeated chest radiograph showed bilateral interstitial pneumonia (Figure 3). The diagnosis was now suspected to be typhus (murine or epidemic) rather than typhoid fever, and the patient was transferred to the intensive care unit, where he was started on intravenous doxycycline, 200 mg/d. His clinical condition improved rapidly, and he became afebrile within 3 days. Doxycycline was stopped after 10 days, when the patient had recovered.

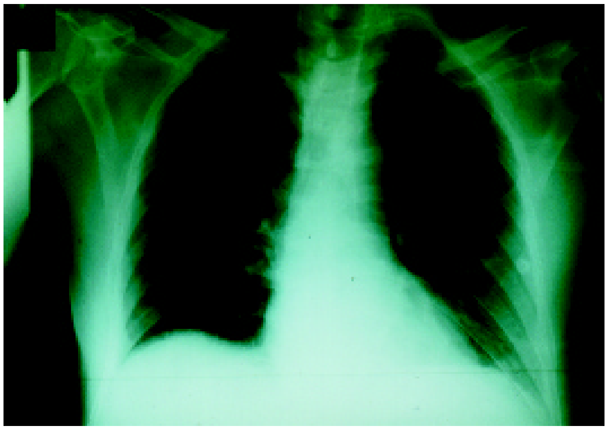

Figure 3. Bilateral interstitial pneumonia (day 7).

The diagnosis of epidemic typhus was established by demonstrating increasing antibody titers from the acute to the convalescent-phase of illness, with the presence of immunoglobulin (Ig) M to *R. prowazekii* (microimmunofluorescence titer IgG <1:80 and IgM <80 to IgG 1:4,096 and IgM titer 1:256) in serum samples collected 6 days apart (4). The diagnosis was confirmed by the isolation of *R. prowazekii* in blood (shell vial technique), followed by polymerase chain reaction identification of specific DNA in blood by using primers reacting with the citrate synthetase gene and verified by gene product sequencing (4).

Because the patient had returned from Algeria, a country where enteric fever is prevalent, the illness was misdiagnosed as typhoid fever, and the patient was treated with ceftriaxone (3). On average, the rash of epidemic typhus appears on day 5 of illness and consists initially of pink macules in the axillary areas, which subsequently spread over the trunk and limbs and eventually become petechial. While petechiae are reported in 33% of patients with typhus, the initial pinkish macules are reported in only 5% of typhus cases (5). Thus, epidemic typhus may be very difficult to distinguish from typhoid fever in the early phase of illness.

Chloramphenicol has been effective against both typhoid fever and typhus. Co-trimoxazole, which may be used to treat typhoid fever, is not effective against rickettsial agents (6), and a patient who was treated with ciprofloxacin for suspected typhoid fever died of typhus (7). The persistently negative blood culture results, combined with the development of petechiae and failure to improve on ceftriaxone, led us to consider the diagnosis of typhus rather than typhoid fever, since rickettsia are resistant to beta-lactam antibiotics (6). The death rate of untreated epidemic typhus is approximately 15%; this rate is reduced to 0.5% with a single 200-mg dose of doxycycline (1). Therefore, epidemic typhus should be considered in the differential diagnosis of typhoid fever, in particular when epidemiologic conditions may have led to contact with body lice.

Typhus has not been reported in Algeria for several decades (8). Because infection is lifelong, humans are regarded as the main reservoir of the bacteria. Recrudescence in the form of Brill-Zinsser disease may act as the source of an outbreak, but wide spread cannot occur without louse infestation, as recent outbreaks in Burundi (1) and Russia (2) have shown. Body lice are prevalent among the homeless in industrialized regions such as Marseilles, France. The importation of typhus by an infected patient could start an outbreak within this exposed population, as has occurred with trench fever (9). Disease surveillance, delousing of exposed persons, and improvement of the living conditions of populations at risk should prevent the reemergence of louse-borne diseases in industrialized countries.

### Acknowledgment

The authors thank J. Bakken for review of the manuscript for English.

Dr. Niang is a fellow in the infectious disease and tropical medicine unit at the Houphouet Boigny University Hospital, Marseilles, France. His fields of interest are rickettsial diseases and bacterial infections.

### References

1. Raoult D, Ndihokubwayo JB, Tissot-Dupont H, Roux V, Faugere B, Abegbinni R, et al. Outbreak of epidemic typhus associated with trench fever in Burundi. Lancet 1998;352:353-8.
2. Tarasevich I, Rydkina E, Raoult D. Outbreak of epidemic typhus in Russia. Lancet 1998;352:1151.
3. Islam A, Butler T, North S. Randomized treatment of patients with typhoid fever by using ceftriaxone or chloramphenicol. J Infect Dis 1988;158:742-7.
4. La Scola B, Raoult D. Laboratory diagnosis of rickettsioses: current approaches to diagnosis of old and new rickettsial diseases. J Clin Microbiol 1997;35:2715-27.
5. Perine PL, Chandler BP, Krause DK, McCardle P, Awoke S, Habte-Gabr E, et al. A clinico-epidemiological study of epidemic typhus in Africa. Clin Infect Dis 1992;14:1149-58.
6. Raoult D, Roux V. Rickettsioses as paradigms of new or emerging infectious diseases. Clin Microbiol Rev 1997;10:694-719.
7. Zanetti G, Francioli P, Tagan D, Paddock CD, Zaki SR. Imported epidemic typhus. Lancet 1998;352:1709.
8. Le Corroller Y, Neel R, Lecubarri R. Le typhus exanthematique au Sahara. Arch Inst Pasteur Alger 1970;48:125-30.
9. Brouqui P, Lascola B, Roux V, Raoult D. Chronic *Bartonella quintana* bacteremia in homeless patients. N Engl J Med 1999;340:184-9.

## References

[PDF_00178] Brouqui P., Lascola B., Roux V., Raoult D. (1999). Chronic Bartonella quintana bacteremia in homeless patients.. N Engl J Med.

[PDF_00161] Islam A., Butler T., Nath S. K., Alam N. H., Stoeckel K., Houser H. B., Smith A. L. (1988). Randomized treatment of patients with typhoid fever by using ceftriaxone or chloramphenicol.. J Infect Dis.

[PDF_00175] Le Corroller Y., Néel R., de Lecubarri R. (1970). Le typhus exanthématique au Sahara.. Arch Inst Pasteur Alger.

[PDF_00155] Raoult D., Ndihokubwayo J. B., Tissot-Dupont H., Roux V., Faugere B., Abegbinni R., Birtles R. J. (1998). Outbreak of epidemic typhus associated with trench fever in Burundi.. Lancet.

[PDF_00170] Raoult D., Roux V. (1997). Rickettsioses as paradigms of new or emerging infectious diseases.. Clin Microbiol Rev.

[PDF_00159] Tarasevich I., Rydkina E., Raoult D. (1998). Outbreak of epidemic typhus in Russia.. Lancet.

[PDF_00173] Zanetti G., Francioli P., Tagan D., Paddock C. D., Zaki S. R. (1998). Imported epidemic typhus.. Lancet.

